# Twin‐Lever Multifunctional Meta‐Optics With High Longitudinal Tolerance

**DOI:** 10.1002/advs.77477

**Published:** 2026-09-16

**Authors:** Isma Javed, Kyungtae Kim, Aqib Raza Shah, Dohyun Kang, Joohoon Kim, Azhar Javed Satti, Xuefeng Liu, Muhammad Zubair, Jichuan Xiong, Muhammad Qasim Mehmood, Junsuk Rho

**Affiliations:** ^1^ School of Electronic and Optical Engineering Nanjing University of Science and Technology Nanjing China; ^2^ Department of Electrical Engineering Information Technology University of the Punjab (ITU) Lahore Pakistan; ^3^ Department of Mechanical Engineering Pohang University of Science and Technology (POSTECH) Pohang Republic of Korea; ^4^ POSTECH Institute of Artificial Intelligence (PIAI) Pohang University of Science and Technology (POSTECH) Pohang Republic of Korea; ^5^ School of Engineering University of Leicester Leicester UK; ^6^ Department of Chemical Engineering Pohang University of Science and Technology (POSTECH) Pohan Republic of Korea; ^7^ Department of Electrical Engineering Pohang University of Science and Technology (POSTECH) Pohan Republic of Korea; ^8^ National Institute of Nanomaterials Technology (NINT) Pohang Republic of Korea; ^9^ POSCO‐POSTECH‐RIST Convergence Research Center for Flat Optics and Metaphotonics Pohang Republic of Korea

**Keywords:** broadband imaging, flat optics, metasurfaces, nanophotonics, polarization control, twin‐lever metalenses

## Abstract

Metasurfaces have transformed nanophotonics by enabling full vectorial control of light's amplitude, phase, and polarization within subwavelength‐thick structures, allowing functionalities unattainable with traditional refractive optics. achieving broad achromaticity and multifunctional operation on one flat platform is still a major challenge for imaging applications using metasurfaces. Additionally, current designs face limitations due to small longitudinal tolerance and aperture size. These issues create strict optical alignment requirements that make practical, real‐time use difficult. Here, we present a twin‐lever design strategy that achieves polarization‐programmable, achromatic broadband dual‐mode imaging (bright‐field focusing and vortex‐mediated edge‐enhanced imaging), with precise control over the axial position of maximum intensity through the aperture, all realized within an ultra‐compact, single‐layer metasurface architecture featuring high longitudinal tolerance. Comprehensive full‐wave simulations and experimental characterizations confirm chromatic‐aberration‐free focusing, spin‐selective phase fidelity, and high‐longitudinal tolerance with aperture‐weighted axial bias across the visible spectrum (488–633 nm). This CMOS‐compatible easy to operate platform establishes a foundation for reconfigurable, multimodal, and broadband optical systems, heralding a new class of intelligent meta‐optical components for next‐generation microscopy, imaging, and integrated photonic technologies.

## Graphical abstract

We introduce a single‐layer, CMOS‐compatible metasurface that allows for polarization‐programmable, achromatic broadband dual‐mode imaging with high longitudinal tolerance. The twin‐lever design combines bright‐field focusing and vortex‐based edge‐enhanced imaging while managing axial intensity distribution from 488 to 633 nm. Simulations and experiments show chromatic‐aberration‐free, spin‐selective performance, creating a compact platform for next‐generation microscopy, imaging, and integrated photonics.

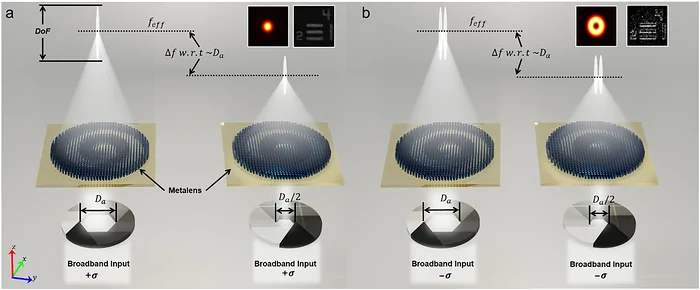

## 1 Introduction

Metasurfaces have become a significant development in contemporary optics, enabling novel ways to manipulate the fundamental properties of light [1, 2, 3, 4, 5]. These two‐dimensional versions of metamaterials, especially those engineered with subwavelength resonant structures, can precisely manipulate the amplitude, phase, and polarization of light [6, 7, 8]. This special engineering of the optical properties enables us to achieve features previously unattainable with traditional naturally occurring materials [9, 10, 11]. The design and implementation of multifunctional achromatic optics are a subject of current research in this relatively new field, offering a viable solution to the long‐term problem of chromatic aberration in advanced optical systems [12, 13, 14, 15].

Chromatic aberration is an inherent issue in many optical systems that arises because different wavelengths of light are bent by different amounts by a material, leading to the different spectral components converging at different points [15, 16]. In the electromagnetic spectrum, this effect causes a significant decrease in resolution, with visible color fringing and defocus blur, resulting in a strong decrease in image quality and system performance [16]. Traditionally, this persistent optical problem has been mitigated by introducing complex refractive systems, often comprising a large number of lens elements fabricated from different optical materials [15, 17]. However, this standard technique automatically increases system complexity, increases manufacturing costs, and contributes considerably to the bulk and weight of optical devices [4, 17].

Even though traditional optical components continue to be used in imaging systems, their inherent disadvantages have led researchers to explore alternative design strategies. In this context, metalenses have attracted significant interest as a potential means of overcoming some of these restrictions [18, 19, 20]. These structures are attractive primarily for practical reasons, including their thin profile, light weight, and compatibility with standard semiconductor fabrication, features that distinguish them from bulky optical components [12, 21, 22]. The working principle of metalenses is not the same as that of traditional refractive lenses. In conventional optical systems, the phase profile is established incrementally as light passes through the lens material. For metalenses, however, the phase response is instead enforced locally, with each subwavelength meta‐atom providing a different phase shift [23, 24]. Localized control of the optical response facilitates wavefront modifications that are challenging to achieve with conventional bulk elements. This capability has been utilized in various designs to mitigate aberrations or to integrate multiple functions into a single optical surface [25, 26]. However, these capabilities are associated with practical and physical trade‐offs, and their advantages are frequently system and wavelength‐dependent. Nevertheless, previous studies demonstrate that metalens‐based designs are suitable for integration into small, compact optical systems and, in certain cases, exhibit broadband achromatic behavior, a feature that is difficult to achieve with conventional refractive lenses [27, 28].

One well‐known method for creating achromatic behavior in metalenses involves designing phase dispersion to counteract wavelength‐dependent focusing effects [6, 15, 29, 30, 31]. In practice, this usually happens by designing meta‐atoms with specific shapes and arrangements. This lets us control the group delay dispersion across a certain bandwidth, so different wavelengths can focus on about the same plane [15, 32]. In the case of flat, diffractive lenses, such fine‐tuning of the phase is especially necessary since chromatic aberrations are more readily perceived. Meta‐atoms designed properly are needed to manage these wavelength‐dependent effects [33, 34]. Moreover, hybrid designs based on metasurfaces with refractive materials have been investigated to provide an additional degree of freedom for chromatic correction, offering wider achromatic bandwidths and higher efficiency than purely diffractive designs [32, 35, 36]. With a combination of refractive and diffractive materials, compact optical systems with large numerical apertures and broader fields of view can be produced, which is essential to high‐performance imaging applications [37, 38]. This kind of integration also allows optical properties to be actively controlled, enabling reconfigurable, adaptable optical systems [39, 40, 41, 42].

Despite significant advances in metalens technology, achieving true achromatic multi‐mode functionality within a single compact architecture remains an unsolved challenge. A fundamental trade‐off persists achromatic metalenses exhibit exceptional spectral fidelity and broadband phase compensation, but are typically limited to single‐mode operation [43, 44, 45, 46, 47]. On the other hand, multifunctional multidimensional metasurfaces, which can polarize multiplex or multimodal images, exhibit functional versatility but suffer from chromatic aberration and operate in narrowband [48, 49].

Efforts to reduce chromatic dispersion tend to increase system complexity or reduce fabrication scalability and efficiency [50, 51]. Even though there has been advancement in dual‐band [52], tunable [53, 54], and polarization‐insensitive [13, 55] devices. Although the field of dispersion‐engineered and multifunctional metasurfaces has advanced rapidly, a proper framework that can unify the principles of achromaticity at broadband frequencies and versatile optical functionality into a single, compact, and fabricable structure has yet to be achieved with state‐of‐the‐art technology [6]. The structural challenges that persist are meta‐atom crosstalk in multi‐wavelength schemes and the complexity of inverse‐design optimization [56, 57]. Overcoming these constraints to simultaneously engineer wavelength‐independent, multifunctional phase control represents the next critical frontier in metalens design. Beyond aberration correction and functional versatility, a persistent challenge is the intrinsically small aperture and short focal length of metalenses. Focal lengths are often only a few hundred micrometers, which complicates integration into optical systems. These short distances make alignment challenging. In imaging applications, where precise focal positioning is critical, even small misalignments can significantly degrade performance. As a result, integration usually requires highly precise optomechanical stages and components, increasing system complexity and constraints.

**FIGURE 1:**
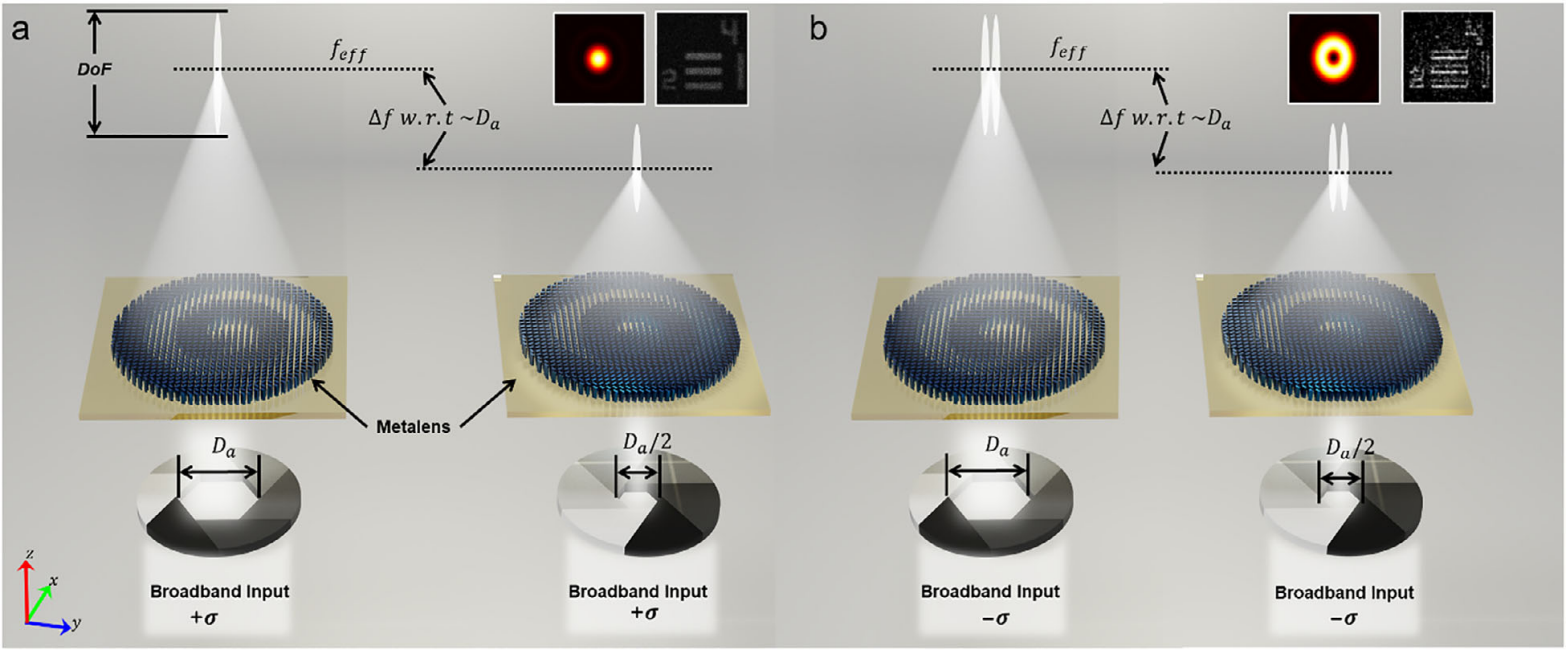
Conceptual schematic of the twin‐lever achromatic, polarization‐programmable multifunctional metalens. The diagram illustrates the dual‐mode achromatic metalens that integrate dispersion‐engineered phase compensation with spin‐dependent geometric‐phase control. (a) Under RCP illumination, the metasurface preserves a wavelength‐independent focal length within the depth‐of‐focus (DoF), enabling broadband achromatic focusing with the axial position of maximum intensity determined by the aperture. (b) Under LCP illumination, the metalens produces a donut‐shaped point‐spread function (PSF), with the axial location of maximum intensity likewise governed by the aperture.

In this work, we present an easy‐to‐integrate twin‐lever design strategy that derives its functionality from two independently controllable mechanisms: polarization‐based control and aperture‐based control as illustrated in Figure 1. The first design lever exploits the polarization‐selective geometric/Pancharatnam‐Berry (PB) phase to realize achromatic, broadband operation in both bright‐field and edge‐enhanced imaging in a single and compact optical platform. The second lever employs aperture engineering to control the axial maximum intensity position by selectively varying the effective aperture, resulting in a controllable longitudinal axial intensity position shift without compromising the achromatic performance. The device harnesses phase engineering to encode two orthogonal optical transfer functions, selectively activated by left‐circularly polarized (LCP) and right‐circularly polarized (RCP) incident states, thereby realizing polarization‐multiplexed image formation without auxiliary optical components. To maintain achromatic performance across the visible spectrum, the metasurface incorporates distributed‐engineered phase compensation, in which the wavelength‐dependent phase response of each meta‐atom counterbalances the chromatic focal shift of the lens profile. Any residual dispersion is confined within the depth of focus, ensuring wavelength‐invariant focusing. This high longitudinal tolerance (large depth of focus) also simplifies alignment in practical implementations. Passive aperture‐weighted axial bias is enabled through distributed phase engineering, which breaks conventional symmetry of the phase mask and allows control over the focal position without introducing additional system complexity. Thus, passive tunable‐aperture metasurface configuration is incorporated to dynamically tailor the spatial‐frequency response, enhancing image contrast and edge definition while preserving broadband achromatic behavior. The device is etched on a CMOS‐compatible substrate with the a‐Si:H material which offers great contrast in the refractive indices and minimal optical losses in the visible band (see Section S1 and Figure S1 of the ellipsometry data). The nano‐resonators are shaped with high‐resolution electron‐beam lithography (EBL) and lead to uniformity across the aperture as well as sub‐wavelength geometric phase encoding. This additive scheme of design and fabrication combines achromatic dispersion control with polarization multiplexing in an unprecedented manner, apparently promising solution to the trade‐off between chromatic aberration correction and polarization multiplexing. This design marks an important step toward polarization‐programmable, broadband‐achromatic metalenses, supporting high‐contrast multimodal microscopy and adaptable photonic imaging systems.

## 2 Design and Methods

In the metasurface twin‐lever design, two independent mechanisms are employed: dispersion‐engineered spin‐decoupled phase modulation and passive aperture‐weighted axial bias. Together, these mechanisms enable broadband achromatic operation while simultaneously providing control over the axial position and polarization‐dependent multifunctionality within a single ultrathin optical platform. The synergy between these two approaches enables a metasurface to pave a path for a unified framework for broadband, tunable planar optics.

### 2.1 Design Principle of the Twin‐Lever Metasurface

When a plane wave illuminates the two‐dimensional metasurface of diameter ***D*** located at ***z***_***o***_ = 0, with the ***z*** − ***x*** plane defining the plane of incidence, the metalens focuses the incident light to a wavelength‐dependent focal length ***f*** (***λ***) as shown in Figure 1a (left). The on‐axis field distribution at an arbitrary observation point ***P*** (0, 0, ***z***) can be described under the Fresnel approximation as:

(1) $$F_{z}\;\left(0,\;0,z\right)=\frac{e^{ikz}}{i\lambda z}\;\int \;\;\;\int_{-D/2}^{D/2}F_{o}\left(x,\;y,\;0\right)\times e^{ik\frac{r^{2}}{2z}}dxdy$$

where $r\;=\sqrt{x^{2}+y^{2}}$ represents the radial coordinates, ***λ*** and ***k*** are the free‐space wavelength and wave number, respectively, and $F_{o}\left(x,y,0\right)$ represents the complex field distribution imparted by the metalens at ***z***_***o***_. The field immediately after the metasurface can be expressed as:

(2) $$F_{o}\;\left(x,\;y,0\right)=\left\{\begin{array}{cc} T\times e^{i\varphi \left(r,\;\lambda \right)} & -\frac{D}{2}<r<\frac{D}{2}\\ 0 & \mathrm{else} \end{array}\right.\;$$

where ***T*** is the transmission amplitude and ***φ*** (***r***, ***λ***) denotes the spatially and spectrally dependent phase profile of the metalens. Thus, Equation (1) can be rewritten as:

(3) $$F_{z}\;\left(0,\;0,z\right)=\frac{Te^{ikz}}{i\lambda z}\;\int \;\;\;\int_{-D/2}^{D/2}e^{ik\left(\varphi \left(r,\;\lambda \right)+\frac{r^{2}}{2z}\right)}dxdy$$

and

(4)

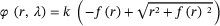

 where ***f*** (***r***) is a radially varying focal length.

#### 2.1.1 Suppression of Chromatic Aberration

For achromatic dispersion engineering, the metalens must suppress variations in the $f\left(r\right)\to \frac{\partial f}{\partial \lambda }\approx 0,$ which is typically scaled linearly with wavelength in diffractive systems. Therefore, the metalens is designed such that the wavelength‐dependent phase dispersion of each meta‐atom compensates for the residual chromatic defocus proportional to $\Delta f\;=\frac{\Delta \lambda }{\lambda }\;\times f$ of the overall lens phase. Thus, the phase ***φ*** (***r***, ***λ***) is optimized by defining focal length ***f*** (***r***) as:

(5) $$f\left(r\right)=\frac{D}{2\tan\left(\sin^{-1}NA_{max}\right)}+f_{dof}\times \left(\frac{2r}{D}\right)$$

subject to the constraint

(6) $$max\left(\Delta f_{@\lambda_{min}},\Delta f_{@\lambda_{max}}\right)<f_{dof}<f_{NA_{min}}$$

where ***NA***_***max***_ is the maximum numerical aperture, chosen as $\frac{2}{\sqrt{5}}$, to ensure susceptible distortion in spectral range. The minimum numerical aperture, ***NA***_***min***_ is designated such that $|\nabla f|<\frac{\lambda_{c}}{{\mathrm{NA}}_{\min}^{2}}|$ which constrains the spatial variation of the focal length. The wavelengths ***λ***_***c***_ ***λ***_***min***_ and ***λ***_***max***_ represent the central, minimum, and maximum wavelengths of the selected spectral band (488–633 nm), respectively. Thus, conditions ensure that residual spectral focal shifts remain within the diffraction‐limited tolerance. Consequently, the metasurface achieves wavelength‐invariant focusing across the visible spectrum without requiring multilayer stacking or refractive compensation with very high longitudinal tolerance. It is worth noting that the observed increase in the depth of focus is not a consequence of a reduced numerical aperture, as is typically the case for conventional lenses. Instead, it is achieved through an engineered longitudinal phase profile while maintaining the same aperture, NA, and focal length. To further illustrate this concept, a numerical comparison between a conventional hyperbolic metalens and the proposed metalens is presented in Section S6. Both metalenses were designed with diameter of 20 µm, an NA of 0.33, a focal length of 28.19 µm, and an operating wavelength of 560 nm. The comparison, presented both qualitatively (Figure S7) and quantitatively (Table S3), demonstrates the effectiveness of the proposed design in achieving an extended lognitudinal tolerance without compromising the lens geometry or optical specifications.

#### 2.1.2 Aperture‐Weighted Axial Bias

For ***D*** ≪ 2 ***f*** (***r***), the phase profile of the metalens can be approximated under the paraxial condition as:

(7) $$\varphi \left(r,\;\lambda \right)\approx -\frac{kr^{2}}{2f\left(r\right)}$$

indicating that the metalens can be interpreted as a superposition of locally quadratic lenses, each characterized by ***f*** (***r***). The on‐axis field at an axial position ***z*** can therefore be expressed using Fresnel diffraction as:

(8) $$F_{z}\;\left(0,\;0,z\right)=\frac{Te^{ikz}}{i\lambda z}\;\int \;\;\;\int_{-D/2}^{D/2}e^{ik\left(\frac{r^{2}}{2z}\times \left(\frac{f\left(r\right)-z}{f\left(r\right)}\right)\right)}dxdy$$

Since the focal length changes with the radius, each ring‐shaped region adds to a different spot along the optical axis. This means that, unlike regular lenses that focus light to a single point, the metalens creates a longer focal segment and breaks the usual symmetry.

Assuming uniform illumination, when a circular aperture of diameter ***D***_***a***_, is introduced, the contributing radial range is truncated to ***r*** < ***D***_***a***_/2. The effective focal position (center of mass of the axial intensity distribution) can then be approximated as:

(9) $$z=f_{min}+f_{dof}\left(\frac{D_{a}}{D}\right)$$

The aperture physically blocks the outer parts of the phase profile, which have larger phase gradients and stronger curvature. This reduces the effect of components with longer focal lengths. Since the phase profile spreads outward from the center, cutting off the edges breaks the symmetry of standard single‐focus metalenses. As a result, the inner curvatures that remain combine at shorter distances along the axis, so the effective focal point moves steadily forward as the aperture gets large. The accessible axial focal range is therefore limited to:

(10) $$f_{min}<z<f_{min}+f_{dof}\times \left(\frac{D_{a}}{D}\right)$$

It is important to emphasize that this aperture‐induced focal shift is fundamentally different from the classical Fresnel focal shift (CFF) [58], where edge diffraction around a stationary quadratic phase profile produces a focal shift that depends on the Fresnel number and a single design focal length (see, Section S3). As shown in Table S1, CFF theory accurately predicts the behavior of conventional metalenses but fails for the present distributed‐phase metalens. This failure arises because the Fresnel number is no longer a meaningful parameter in the absence of a unique stationary phase focus. Instead, the aperture modifies the relative weighting of multiple axial focal contributions, breaking the symmetry inherent to single‐focus designs and giving rise to the observed aperture‐dependent axial profile redistribution.

#### 2.1.3 Polarization‐Selective Dual‐Mode Operation

After establishing achromatic focusing and focal position control, the metasurface exploits polarization‐dependent phase modulation to realize dual optical functionality within the same aperture. Accordingly, the spin sensitivity of the anisotropic nano‐resonators is designed so that each element acts as a local birefringent unit, giving opposite phase shifts when illuminated with orthogonal circularly polarized (CP) light. The Jones matrix of such an anisotropic nano‐resonator $J_{anr}$ can be written as [59]:

(11) $$J_{anr}=\eta_{gs}\;\times \left[\;\begin{array}{cc} 1 & 0\\ 0 & 1 \end{array}\;\right]+\eta_{anr}\times \left[\;\begin{array}{cc} \cos^{2}\theta & \sin\theta \cos\theta \\ \sin\theta \cos\theta & \sin^{2}\theta \end{array}\;\right]$$

where ***η***_***gs***_ and ***η***_***anr***_ denote the conversion efficiency coefficients associated with the glass substrate and the anisotropic nano‐resonators, respectively, while ***θ*** represents the in‐plane orientation angle of the anisotropic nano‐resonators. Under the illumination of the CP, the transmitted light can be expressed as the superposition of the orthogonal components of the CP as:

(12) $$\begin{array}{c} J_{anr}|C\;P^{\pm \sigma }=\left(\eta_{gs}\;\times |CP^{\pm \sigma }\right)+\eta_{anr}\times \frac{1}{2}\left(\left|CP^{\pm \sigma }+e^{\pm i2\theta }\right|CP^{\mp \sigma }\right) \end{array}$$

where − ***σ*** and + ***σ*** denote the left‐ and right‐handed CP states, respectively. Upon transmission through the metasurface, the optical field is decomposed into a co‐polarized component, which preserves its original polarization state, and a cross‐polarized component, whose handedness is reversed and which acquires an additional geometric phase shift of ± 2 ***θ***. This phase originates from the in‐plane rotation of the anisotropic nano‐resonators rather than from propagation delay and is therefore inherently wavelength‐scalable and weakly sensitive to material dispersion. Consequently, a full 0 to 2 ***π*** phase coverage can be achieved by appropriately rotating the nano‐resonators to satisfy the required phase profile ***φ*** (***r***, ***λ***). The local orientation angle of each nano‐resonator is given by ***θ*** (***r***) = ***φ*** (***r***, ***λ***)/2. When both spin states are simultaneously present, the transmitted field arises from the coherent superposition of the two conjugate spin channels, yielding the total phase:

(13)

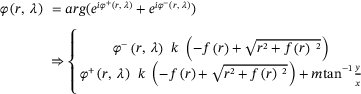

 where ***φ*** ^+^(***r***, ***λ***) and ***φ*** ^−^(***r***, ***λ***) represent the phase profiles for right‐ and left‐circularly polarized light, respectively, while the additional spiral phase term $m\tan^{-1}\frac{y}{x}$ introduces a helical wavefront carrying orbital angular momentum (OAM) of topological charge ***m***. This spin‐decoupled approach allows separate control of wavefronts for each polarization state. RCP illumination leads to broadband focusing, while LCP illumination creates a vortex phase that enhances image edges. By combining dispersion‐engineered achromaticity with polarization‐multiplexed geometric‐phase control, the metasurface becomes a passive, reconfigurable, and broadband optical platform that works reliably across the visible spectrum.

### 2.2 Optimization of the Anisotropic Nano‐Resonator

**FIGURE 2:**
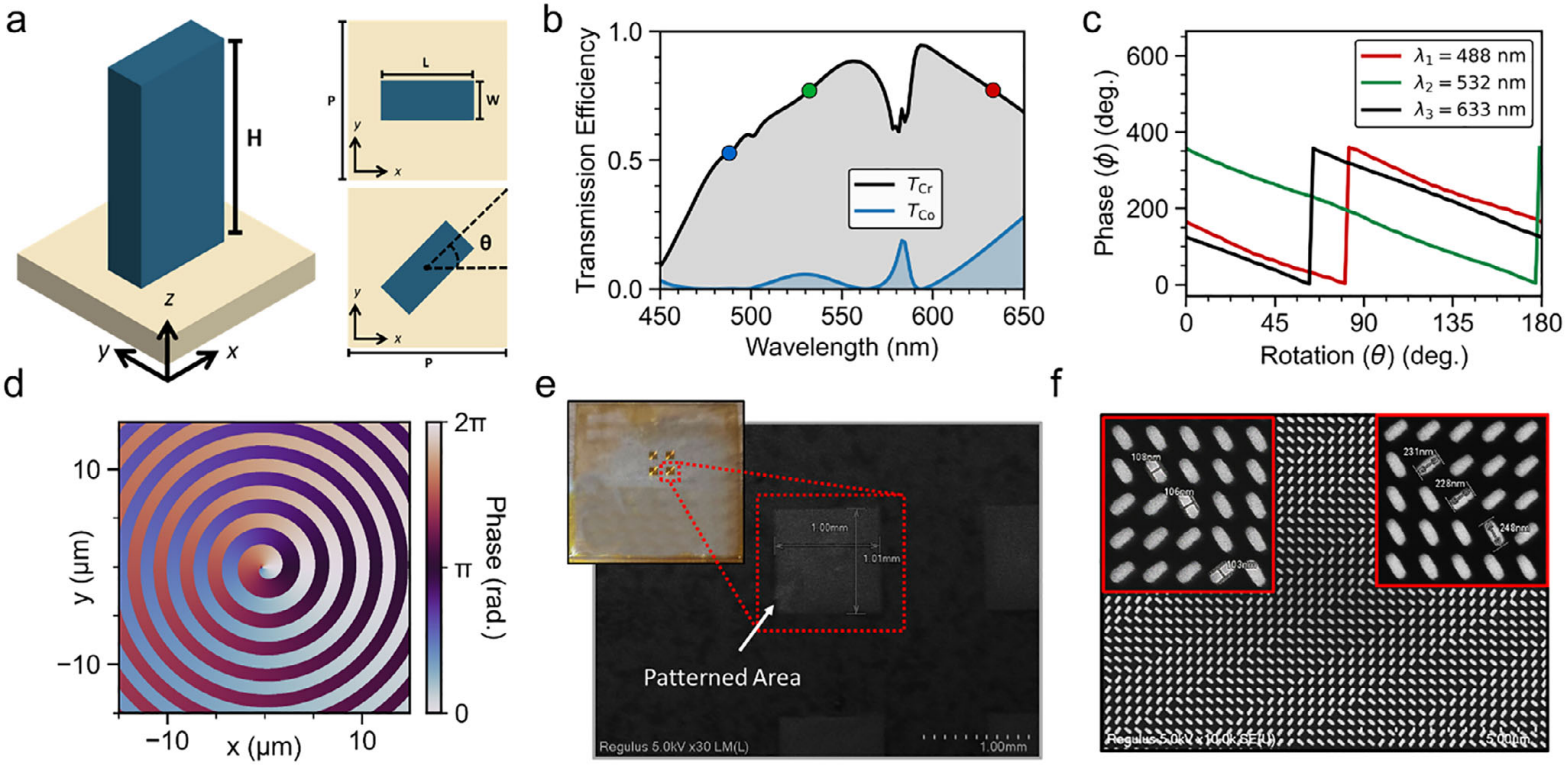
Design and fabrication of the twin‐lever metalens. (a) A schematic illustration of a nano‐resonator functioning as half waveplate (HWP); the phase distribution from 0 to 2π is produced by rotating the nano‐antenna around the *x*‐axis, with θ denoting the rotational angle. (b) The broadband polarization conversion response of the nano‐resonator shows a polarization conversion efficiency exceeding 80% across the entire visible spectrum. (c) The phase coverage changes from 0 to 2π as the angle rotates. (d) The proposed twin‐lever designed phase mask. (e) The patterned area. (f) SEM images of the fabricated twin‐lever metalens sample.

The metasurface design began with optimizing the geometry of the basic nano‐resonator. Its main structural parameters; length (L), width (W), and lattice period (P) are shown in Figure 2a. The process started by adjusting the lattice period P to achieve efficient spatial sampling of the rotational phase distribution and to reduce higher‐order diffraction modes. By analyzing the polarization conversion response as a function of the rotation angle θ, an optimal periodicity of P = 290 nm was identified, providing the highest cross‐polarization efficiency with negligible diffractive leakage.

Following this, a comprehensive parametric sweep of the in‐plane dimensions L and W was conducted to maximize the cross‐polarized transmission (*T_cr_*) while minimizing the co‐polarized transmission (*T_co_* ​) across the visible spectrum. This optimization ensures that the metasurface operates primarily in the geometric‐phase regime, where the transmitted wavefront is dictated by the orientation‐dependent PB phase of the anisotropic nano‐resonators. The optimization workflow used to derive these parameters are detailed in Section S2, where Rigorous Coupled‐Wave Analysis (RCWA) framework are presented. Utilizing the optimized nano‐resonator geometry (L = 230 nm, W = 90 nm, P = 290 nm), a spatially varying phase mask was constructed to experimentally validate the design methodology.

Full‐wave electromagnetic simulations were subsequently performed using a Finite‐Difference Time‐Domain (FDTD) solver to evaluate the metasurface's broadband performance. The results confirmed that the optimized configuration enables high‐efficiency polarization conversion (Figure 2b) and continuous 0→2π geometric‐phase modulation across the visible band (Figure 2c). Simulations of electric‐field intensity and spectral transmission, shown in Section S2, demonstrate that resonant modes are strongly confined and phase coverage is uniform across the operating wavelengths. Figure 2d shows the phase profile of the optimized metasurface, which closely matches the targeted theoretical phase distribution.

After numerical analysis, we fabricated the metasurface using standard EBL, followed by film deposition and lift‐off process to create the periodic array of nano resonators. The fabricated metasurface metalens, patterned over an area of 1 mm × 1 mm, exhibits excellent structural precision and optical quality. The optical image in Figure 2e shows the uniformly patterned region on the SiO_2_ substrate, while the corresponding Scanning‐electron‐microscopy (SEM) micrographs in Figure 2f reveal well‐defined nano‐resonator morphology, accurate rotational alignment, and high uniformity across the entire aperture. The fabricated structures exhibit a strong one‐to‐one correspondence with the designed phase distribution confirming precise realization of the targeted metasurface profile. These results verify that the lithographically fabricated a‐Si:H nano‐resonators accurately reproduce the intended geometric‐phase modulation and maintain high dimensional fidelity and pattern integrity across the large‐area device.

## 3 Results and Discussion

### 3.1 Simulation and Optical Characterization

To evaluate the achromatic dual‐mode operation with axial position control of the proposed metalens, full‐wave electromagnetic simulations were performed using the final phase mask defined by Equations (13). The formulation encodes the spatially varying phase, where the in‐plane rotation of each anisotropic meta‐atom imposes a spin‐selective phase retardation. By embedding the required phase into the orientation of the nanostructures, a single metasurface is engineered to support two independent optical functionalities controlled solely by the spin state of incident light.

**FIGURE 3:**
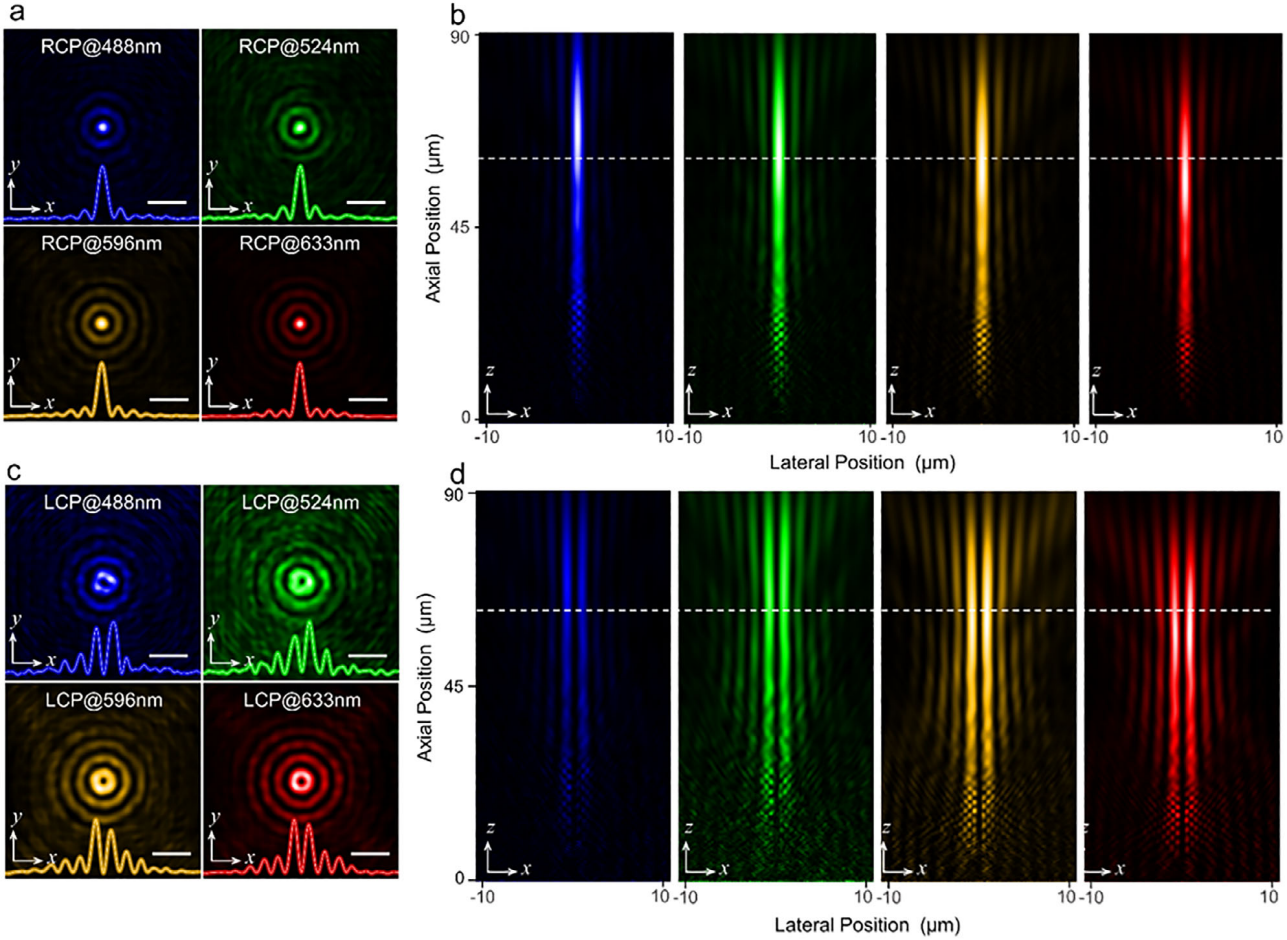
Simulated broadband achromatic dual‐mode operation of the proposed metalens (full aperture illumination). (a,c) Transverse focal‐plane intensity distributions (*x‐y* plane) under RCP and LCP illumination, respectively, shown over a 10 µm × 10 µm field of view (scale bars = 1 µm). RCP light produces a diffraction‐limited Airy PSF, while LCP light generates a doughnut‐shaped focal spot with a 2 ***π*** azimuthal phase accumulation, characteristic of a first‐order OAM mode. (b,d) Corresponding longitudinal cross‐sections along the optical axis (*x‐z* plane, 10 µm × 90 µm), demonstrating tightly confined focal waists and well‐defined wavefront convergence. The focal positions remain essentially invariant across the visible spectrum (488, 524, 596, 633 nm), confirming achromatic focusing behavior.

As shown in Figure 3, the designed 40 µm × 40 µm metasurface exhibits robust broadband dual‐mode behavior across the visible spectrum (488, 524, 596, and 633 nm) with full aperture illumination (*i.e*. *D_a_* = *D*). Under RCP illumination, the metalens produces a diffraction‐limited converging wavefront, forming a conventional Airy point‐spread function (PSF). In contrast, LCP illumination triggers a vortex phase profile with a 2π azimuthal phase accumulation, yielding a doughnut‐shaped focal intensity distribution with an on‐axis null, a signature of a first‐order OAM mode (*m* = ± 1). This polarization‐dependent helical phase confirms precise spin‐controlled geometric‐phase modulation at the subwavelength scale.

The transverse focal‐plane (maximum intensity position) ***z*** = 61.125 **µm** shown in Figure 3a,c are displayed over a 10 µm × 10 µm field of view, capturing the complete PSF within the plotted window. The longitudinal cross‐sections shown in Figure 3b,d, spanning 10 µm × 90 µm, reveal a tightly confined focal width and distinct wavefront convergence, confirming high longitudinal tolerance. The focal position remains nearly the same across all wavelengths, indicating that the lens is achromatic within its operating range. Section S4 (Figures S3 and S4) shows the intensity distributions for the dual‐mode achromatic metalens when the effective aperture is reduced to ***D***_***a***_ = 3 ***D***/4, and ***D***/2. When the aperture is narrowed, the PSF broadens and the point of maximum intensity shifts. This shows that focusing is mainly governed by the locally tuned phase gradient and the overall aperture size, enabling precise control of the metalens axial intensity. Table S2 (Section S3) summarizes all focal‐position measurements for the different aperture cases at a wavelength of 633 nm. Because the phase profile is distributed to flatten the axial intensity, Table S2 presents both predicted and measured values that approximate the center of mass of the axial intensity distribution. The predicted positions show good agreement with the simulated results, with deviations of less than 10% from predicted.

### 3.2 Fabrication and Characterization of Metalens

To experimentally validate the achromatic dual‐mode response, a 1*mm* metalens with *f_min_* = 0.250 mm and *f_dof_* ≈ 1.1 mm was fabricated using high‐resolution EBL on a‐Si:H layer deposited via PE‐CVD on a SiO_2_ substrate. The designed phase distribution was patterned into the resist, transferred into the a‐Si:H layer using a Cr hard mask and reactive ion etching, and then exposed as vertically aligned nanopillars with precise orientation and aspect ratio. SEM confirms the fabricated metasurface exhibits uniform meta‐atom distribution with precise orientation, periodicity, and dimensional accuracy, accurately realizing the designed geometric‐phase profile, detailed fabrication workflow is provided in, Section S5 and Figure S5. The achromatic performance of the fabricated metalens was experimentally validated using a custom‐built optical characterization setup (Figure S6).

**FIGURE 4:**
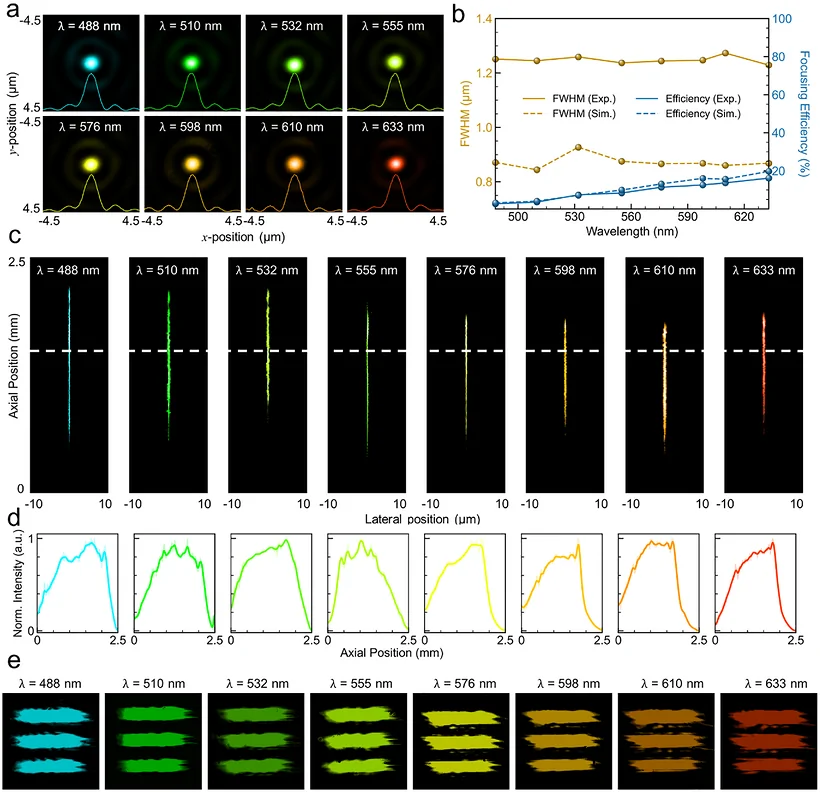
Experimental characterization and imaging performance of the fabricated achromatic dual mode metalens with full aperture (D_a_ = D) RCP illumination. (a) Experimentally measured focal spot intensity distributions in the focal plane (*x‐y*) at 488, 510, 532, 555, 576, 598, 610, and 633 nm, across the visible spectrum. Insets show the corresponding cross‐sectional intensity profiles. (b) Measured and simulated full width half maximum (FWHM) and focusing efficiency as a function of wavelength. (c) Longitudinal (*x‐z*) intensity distributions at the corresponding wavelengths, illustrating tightly confined focal spots with nearly identical axial focal positions over the visible range. (d) Normalized axial intensity profiles extracted from the longitudinal intensity maps in (c), confirming wavelength‐independent focal positions and consistent axial focusing characteristics. (e) Experimental imaging results obtained fixed focal plane at each wavelength, demonstrating broadband brightfield image formation and consistent spatial resolution across the visible spectrum.

Figure 4a presents the experimentally measured transverse (*x* − *y*) focal‐plane intensity distributions of the achromatic metalens at 488, 510, 532, 555, 576, 598, 610, and 633 nm under full‐aperture RCP illumination. The corresponding normalized one‐dimensional intensity profiles extracted along the indicated transverse direction are shown as insets. At all measured wavelengths, the focal spots exhibit well‐defined central maxima surrounded by concentric Airy rings, confirming high phase uniformity across the aperture, and effective suppression of chromatic aberration throughout the visible spectrum.

Figure 4b shows a comparison of the measured and simulated FWHM and focusing efficiency across different wavelengths. The simulations predict diffraction‐limited focusing values (0.84–0.93 µm) across the entire operating range. However, the experimentally measured focal spots are slightly broader, with FWHM of approximately 1.24–1.28 µm. Furthermore, because the lens exhibits an extended depth of focus, the focal plane in both simulations and experiments is defined as the axial position of maximum intensity, and the FWHM is measured at this plane. In the experimental measurements, however, the intensity distribution may not be perfectly uniform due to measurement noise, alignment errors, and other experimental uncertainties. Consequently, the experimentally identified maximum‐intensity plane may deviate from the ideal focal position, resulting in discrepancies in the measured FWHM. Moreover, focusing efficiency was calculated by dividing the optical power within the focal spot by the total incident optical power. As shown in Figure 4b, the fabricated metalens achieves a maximum focusing efficiency of 19% at 633 nm, while maintaining stable broadband performance across the visible wavelength range. The achromatic behavior was further investigated by measuring the longitudinal (*x‐z*) intensity distributions, as shown in Figure 4c. The results reveal tightly confined focal spots with nearly identical axial focal positions from 488 to 633 nm, demonstrating that the focal length remains essentially invariant with wavelength. The corresponding normalized axial intensity profiles shown in Figure 4d further confirm the overlap of the focal maxima, providing strong experimental evidence of broadband achromatic focusing with minimal longitudinal chromatic aberration.

**FIGURE 5:**
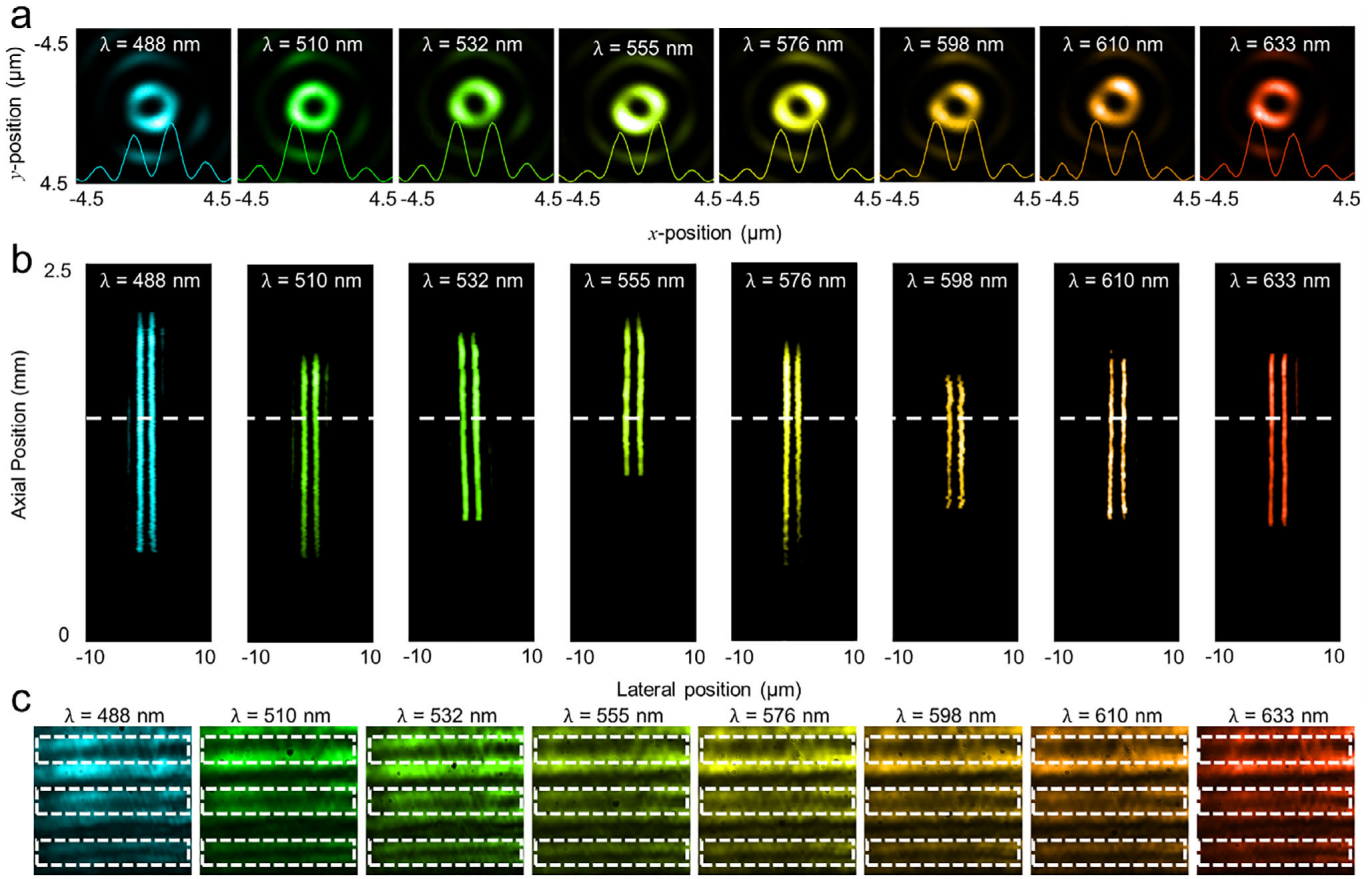
Experimental characterization and imaging of the fabricated achromatic dual mode metalens with full aperture (D_a_ = D) LCP illumination. (a) Experimentally measured focal spot intensity distributions in the focal plane (*x‐y*) at 488, 510, 532, 555, 576, 598, 610, and 633 nm, across the visible spectrum. (b) Longitudinal (*x‐z*) intensity distributions at the corresponding wavelengths, tightly confined focal waists and well‐defined wavefront convergence. (c) Experimental imaging results obtained fixed focal plane at each wavelength, demonstrating broadband edge‐enhances image formation.

In contrast, LCP illumination (Figure 5a) produces a doughnut‐shaped focal‐intensity distribution corresponding to a first‐order optical vortex mode (*m* = ± 1), characterized by a pronounced on‐axis null and azimuthal phase singularity. The associated normalized transverse profiles (Figure 5b) exhibit symmetric twin lobes, consistent with the vortex nature of the focal field and indicating robust spin‐controlled wavefront shaping.

In both polarization channels, the focal positions remain invariant with wavelength, validating the broadband achromatic response of the geometric‐phase twin‐level metalens. This wavelength‐independent focusing behavior, combined with distinct spin‐dependent focal‐field profiles, demonstrates reliable polarization‐multiplexed multifunctionality with consistent performance across the visible spectrum.

Using the experimental setup shown in Figure S6c, The imaging performance of the proposed achromatic twin‐lever metalens was experimentally characterized using a 1951 USAF resolution target. To assess its broadband achromatic capability, images of Group 5, Element 4 (RCP) and Group 4, Element 2 (LCP) were acquired at a fixed image plane under illumination wavelengths of 488, 510, 532, 555, 576, 598, 610, and 633 nm, covering the visible spectrum. For all investigated wavelengths, under LCP illumination (Figure 5c) yields an edge‐enhanced representation characterized by amplified high‐spatial‐frequency features, whereas under RCP illumination (Figure 4e) produces a bright‐field‐like image dominated by low‐spatial‐frequency content and overall intensity contrast. This behavior arises from the distinct spatial structures of the polarization‐dependent PSFs. It is important to note that the imaging resolution achieved at an individual wavelength is slightly lower than that of a conventional monochromatic metalens optimized for a single wavelength. Additionally, owing to the extended depth of focus, the imaging system becomes more sensitive to alignment within the limited field of view (FOV). Consequently, slight misalignments can affect the imaging performance, and only the focal region is presented in the imaging analysis. However, the proposed metalens successfully achieves broadband achromatic imaging across the visible spectrum while maintaining a fixed focal plane. This capability represents a significant advantage over conventional monochromatic designs and makes the proposed metalens a promising candidate for a wide range of practical imaging applications.

**FIGURE 6:**
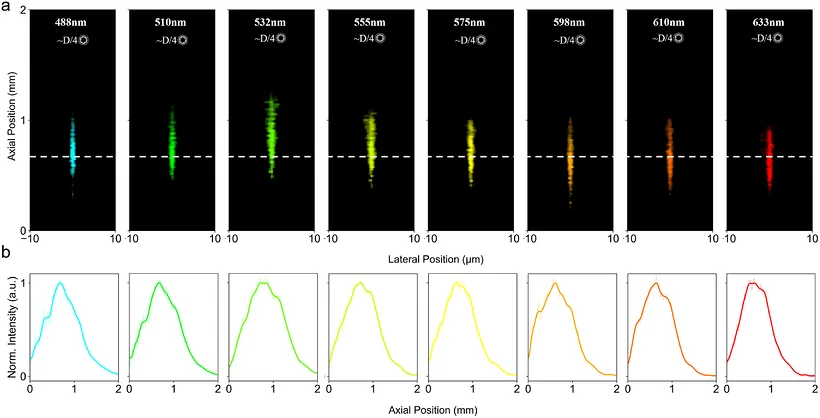
Axial intensity profiles for truncated‐aperture analysis of the twin‐lever metalens. (a) Longitudinal (*x‐z* plane) intensity distributions at 488, 510, 532, 555, 575, 598, 610, and 633 nm, illustrating the focal spot characteristics under a reduced aperture (*D_a_* = *D*/4). (b) Corresponding normalized axial intensity profiles extracted from the longitudinal intensity distributions, showing wavelength‐dependent focal intensity distributions and the axial position of the maximum intensity. The reduced aperture results in a broader point spread function (PSF), increased side‐lobe intensity, and a shift of the maximum intensity position toward the metalens.

As the proposed twin‐lever design enables aperture‐based control of the axial focal‐plane position, its effectiveness was further validated under an extreme aperture condition in which the effective aperture was reduced to one‐quarter of its original size. The focusing performance of the metalens was then characterized to evaluate its ability to maintain the designed focal‐plane position despite the significant reduction in aperture. The optical setup used for the variable‐aperture characterization is shown in Figure S6b. Figure 6 presents measured focusing characteristics of the metasurface metalens under the reduced‐aperture condition (*D_a_* = *D*/4) illustrating how aperture truncation influences the axial focusing behavior.

Figure 6a shows the broadband longitudinal (*x* − *z*) intensity distributions at 488, 510, 532, 555, 575, 598, 610, and 633 nm. Compared with the full‐aperture case, aperture truncation results in a noticeable shift of the focal position toward the metalens, accompanied by a reduction in the longitudinal focal depth which is well aligned with theoratical and numaerical predictions. Despite these changes, well‐confined focal spots are maintained across the entire visible spectrum, demonstrating that the metasurface preserves efficient broadband achromatic focusing under a significantly reduced effective aperture. The observed focal shift arises from geometric aperture truncation while the numerical aperture remains fixed, indicating that the focusing behavior is primarily determined by the locally encoded geometric‐phase distribution rather than the overall pupil geometry. Figure 6b presents the corresponding normalized axial intensity profiles extracted from the longitudinal intensity maps. The profiles reveal broader focal distributions, characterized by an increased FWHM and more pronounced side lobes, consistent with the expected degradation in axial resolution under reduced‐aperture illumination. Nevertheless, each wavelength exhibits a distinct and well‐defined intensity maximum, confirming robust and continuous control over the axial position of the maximum intensity across the operating wavelength range.

## 4 Conclusion

The twin‐lever metasurface design we developed is a major step forward for practical broadband achromatic and multifunctional flat optics with passive controls. Earlier achromatic metalenses often used multilayer dispersion compensation, propagation‐phase hybridization, or complex meta‐atom stacking, which made them harder to fabricate and less efficient. In contrast, our design delivers broadband spectral and functional performance in a single flat structure by using geometric‐phase‐based dispersion engineering. (see Table S3 in Section S6). The measured results confirm wavelength‐invariant focal stability and polarization‐selective dual‐mode operation, validating the dispersion‐compensated phase encoding strategy. Moreover, controlling the aperture size enables axial position tuning, while the large longitudinal depth adds robustness and makes the system easy to align. The close agreement between simulated and experimental PSFs across multiple wavelengths verifies high phase fidelity and subwavelength fabrication precision. With its CMOS‐compatible, single‐step fabrication and scalable planar configuration, this approach is readily transferable to wafer‐scale production and integration with existing photonic platforms. The demonstrated performance establishes a technically viable and manufacturing‐ready pathway toward miniaturized, polarization‐programmable, broadband easy‐to align meta‐optical systems for advanced imaging, sensing, and integrated photonic applications.

## 5 Experimental Section

### 5.1 Metalens Fabrication

The fabrication of the metalens began with depositing a 400 nm thick a‐Si layer onto a 500 µm‐thick fused silica substrate using plasma‐enhanced chemical vapor deposition (PECVD), with SiH_4_ and H_2_ gas flow rates of 10 and 75 sccm respectively with temperature of 100°C and pressure of 30 mTorr. After spin‐coating a positive‐tone photoresist (Microchem, PMMA 495 A2) and a conductive polymer layer (Showa Denko, E‐spacer 300Z), electron beam lithography was then employed to pattern the metalens. After exposure, the photoresist was developed for 10 min in a solution of isopropyl alcohol (IPA) and methyl isobutyl ketone (MIBK) at a 1:3 ratio. An electron‐beam evaporation deposited a 40 nm chromium (Cr) layer as an etching mask. Subsequently, the a‐Si:H layer was etched via a dry etching process, and the Cr mask was removed using CR‐7 etchant.

### 5.2 Metalens Characterization and Imaging

Broadband coherent illumination was generated by a supercontinuum laser and spectrally filtered using an LLTF bandpass filter at 488, 510, 532, 555, 576, 598, 610, and 633 nm, corresponding to the wavelengths used in simulations. The Incident polarization states were precisely prepared using a linear polarizer (LP, LPVISE050‐A, Thorlabs) followed by an achromatic quarter‐wave plate (QWP, AQWP05M‐600, Thorlabs), enabling selective illumination with of LCP or RCP light.

For metalens characterization, the light transmitted through the metalens was collected by an objective lens (MPlanFL N 100×, Olympus) coupled with a tube lens (TTL180‐A, Thorlabs) to relay the focal plane onto a sCMOS camera (pco.panda 4.2 bi UV sCMOS camera, Excelitas Technologies Corp.). Unconverted light was filtered out using a polarization filter consisting of a QWP and an LP placed in the relay system. The relay system and sCMOS camera were mounted on a motorized stage to precisely investigate the intensity distribution of the metalens along the optical axis. Transverse and axial intensity mapping were performed to obtain volumetric scans of the metalens, with background subtraction and flat‐field correction applied to ensure quantitative fidelity of the measured intensity distributions.

For metalens imaging application, light collected by an objective lens (UPlanFL N 10×, Olympus) passed through the USAF resolution target. The resulting scattered light was subsequently collected by the metalens and relayed onto the sCMOS camera through a tube lens. Unconverted light was filtered out using a polarization filter consisting of a QWP and an LP.

## Author Contributions

**Isma Javed**: investigation, methodology, writing – original draft, formal analysis, data curation. **Kyungtae Kim**: investigation, methodology, writing – original draft, formal analysis, data curation. **Aqib Raza Shah**: methodology, investigation. **Dohyun Kang**: investigation, methodology. **Joohoon Kim**: investigation, methodology. **Azhar Javed Satti**: investigation, methodology. **Xuefeng Liu**: investigation, methodology. **Muhammad Zubair**: methodology, supervision, project administration. **Jichuan Xiong**: supervision, project administration. **Muhammad Qasim Mehmood**: writing – review and editing, conceptualization, funding acquisition, project administration, supervision, validation. **Junsuk Rho**: writing – review and editing, conceptualization, funding acquisition, supervision, project administration, resources, validation.

## Conflicts of Interest

The authors declare no conflicts of interest.

## Supporting information




**Supporting File 1**: advs77477‐sup‐0001‐SuppMat.docx.

**Supporting File 2**: advs77477‐sup‐0002‐SuppMat.pdf.

## Data Availability

The data that support the findings of this study are available from the corresponding author upon reasonable request.
