# Supplementary material for: Twin‐Lever Multifunctional Meta‐Optics With High Longitudinal Tolerance
Source: Adv Sci (Weinh). 2026 Sep 16:e77477. Online ahead of print. doi: 10.1002/advs.77477 (PMC13583116; doi:10.1002/advs.77477)
Supplement: Supplementary file 1 [file ADVS-9999-e77477-s001.pdf]

Supporting Information

Twin-Lever Multifunctional Meta-Optics with High Longitudinal Tolerance

Isma Javed†, Kyungtae Kim†, Aqib Raza Shah†, Dohyun Kang†, Joohoon Kim, Azhar Javed Satti, Xuefeng Liu, Muhammad Zubair, Jichuan Xiong*, Muhammad Qasim Mehmood*, and Junsuk Rho**

Isma Javed, Xuefeng Liu, Jichuan Xiong
School of Electronic and Optical Engineering, Nanjing University of Science and Technology, Nanjing 210094, China
*Email: jichuan.xiong@njust.edu.cn

Kyungtae Kim, Dohyun Kang, Joohoon Kim, Junsuk Rho
Department of Mechanical Engineering, Pohang University of Science and Technology (POSTECH), Pohang 37673, Republic of Korea
*E-mail: jsrho@postech.ac.kr

Isma Javed, Aqib Raza Shah, Azhar Javed Satti, Muhammad Qasim Mehmood
Department of Electrical Engineering, Information Technology University of the Punjab (ITU), Lahore 54000, Pakistan
*E-mail: qasim.mehmood@itu.edu.pk

Joohoon Kim
POSTECH Institute of Artificial Intelligence (PIAI), Pohang University of Science and Technology (POSTECH), Pohang 37673, Republic of Korea

Muhammad Zubair
School of Engineering, University of Leicester, Leicester LE1 7RH, United Kingdom
*E-mail: muhammad.zubair@leicester.ac.uk

Junsuk Rho
Department of Chemical Engineering, Pohang University of Science and Technology (POSTECH), Pohang 37673, Republic of Korea
Department of Electrical Engineering, Pohang University of Science and Technology (POSTECH), Pohang 37673, Republic of Korea
National Institute of Nanomaterials Technology (NINT), Pohang 37673, Republic of Korea
POSCO-POSTECH-RIST Convergence Research Center for Flat Optics and Metaphotonics, Pohang 37673, Republic of Korea

† These authors have contributed equally to this work.

Section I: Material ellipsometry data

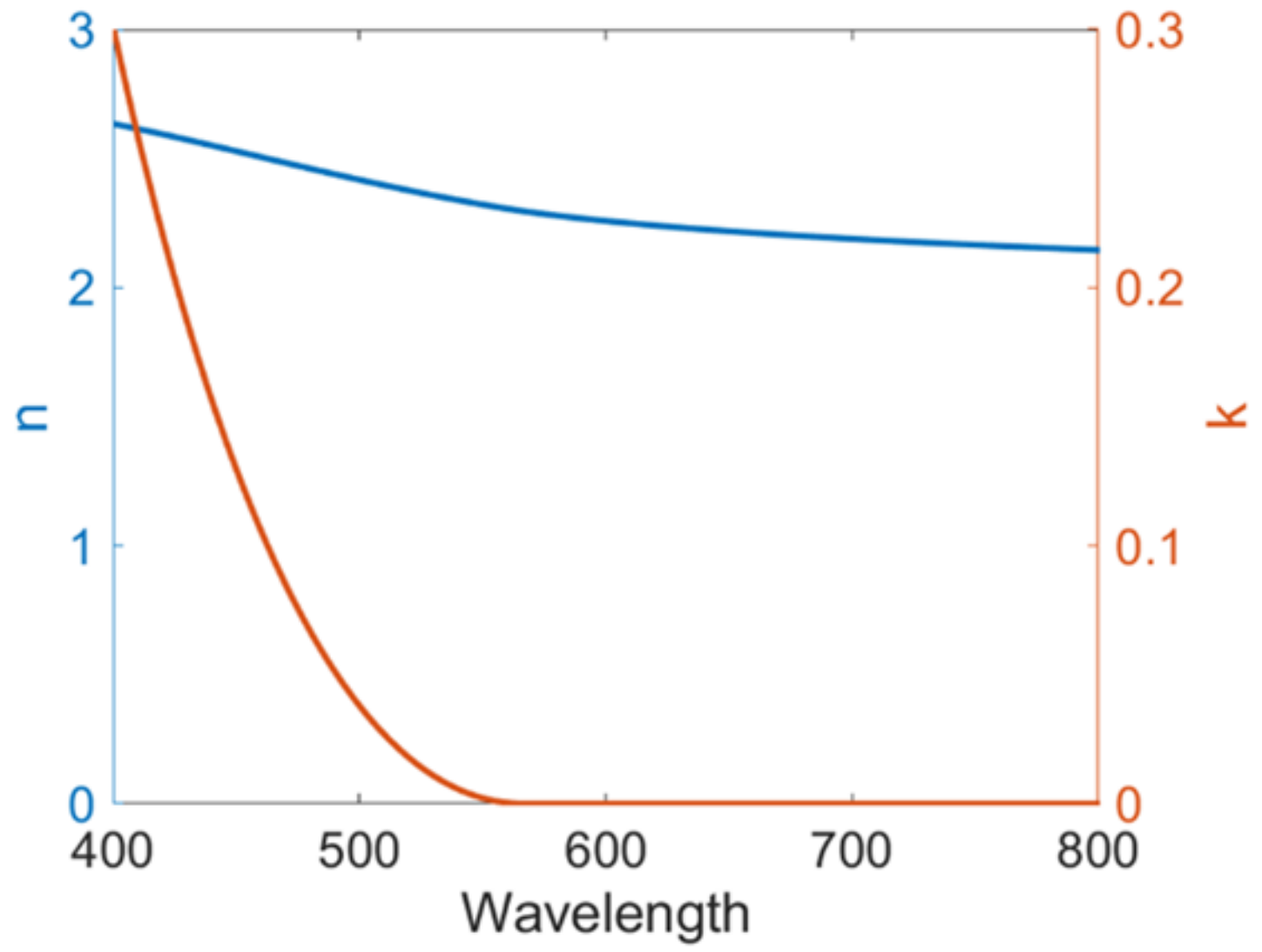

Figure S1. Measured refractive index (n) and extinction coefficient (k) of a-Si:H in the visible range using spectroscopic ellipsometry. The complex refractive indices are determined to be 2.53 + 0.13i, 2.35 + 0.01i, and 2.23 + 0.00i at wavelengths of 450 nm, 532 nm, and 635 nm, respectively.

Section II: Meta-atom optimization

Polarization-Dependent Phase Manipulation in the Visible Spectrum

To achieve efficient polarization-dependent phase manipulation across the visible spectrum, we designed a metasurface composed of anisotropic nano-resonators patterned from hydrogenated amorphous silicon (a-Si:H) deposited on a SiO_2 substrate. The incorporation of hydrogen (H) in a-Si:H passivates dangling bonds and suppresses mid-gap defect states inherent to amorphous silicon. This substantially reduces optical absorption losses and enhances transparency throughout the visible range, as verified in Figure S1.

To suppress spatial aliasing and maintain high-fidelity sampling of the desired phase profile, the in-plane lattice period (P) was designed according to the Nyquist sampling criterion, ensuring

$P < \frac{\lambda}{2NA}$. This prevents the emergence of higher-order diffraction modes under high-NA focusing conditions. A parametric sweep of $P = 250 - 350\, nm$ (step size 2 nm) was conducted while monitoring the polarization conversion efficiency as a function of the rotation angle θ. The optimal periodicity of $P = 290\, nm$ was selected based on the highest cross-polarized efficiency and effective suppression of spurious diffraction orders (Figure S2g).

The structural height (H) of the nano-resonators was fixed at 400 nm, chosen to ensure strong electromagnetic confinement and efficient excitation of resonances across the visible band. Subsequent Rigorous Coupled-Wave Analysis (RCWA) simulations systematically varied the nano-resonator length (L) and width (W) within the range of $50 - 250\, nm$, generating a comprehensive geometric library. The resulting dataset was analyzed to identify configurations providing (i) uniform $0 \rightarrow 2\pi$ phase coverage in the cross-polarized channel and (ii) strong polarization conversion with minimal co-polarized leakage across 488 nm, 532 nm, and 633 nm. The optimal configuration $L = 230\, nm$ and $W = 90\, nm$ was selected by mapping the retrieved complex transmission coefficients of the cross-polarized (Figures S2a-c) and co-polarized components (Figures S2d-f) respectively.

At the optimized geometry, the x–z cross-sectional electric field intensity distributions ($|E|^2$) reveal distinct resonance modes responsible for polarization-selective behavior. As shown in Figures S2h and S2i, the field maps for TE and TM excitations exhibit pronounced confinement within the nano-resonator volume, confirming the excitation of resonances that underlie the observed high-efficiency polarization conversion and broadband geometric.

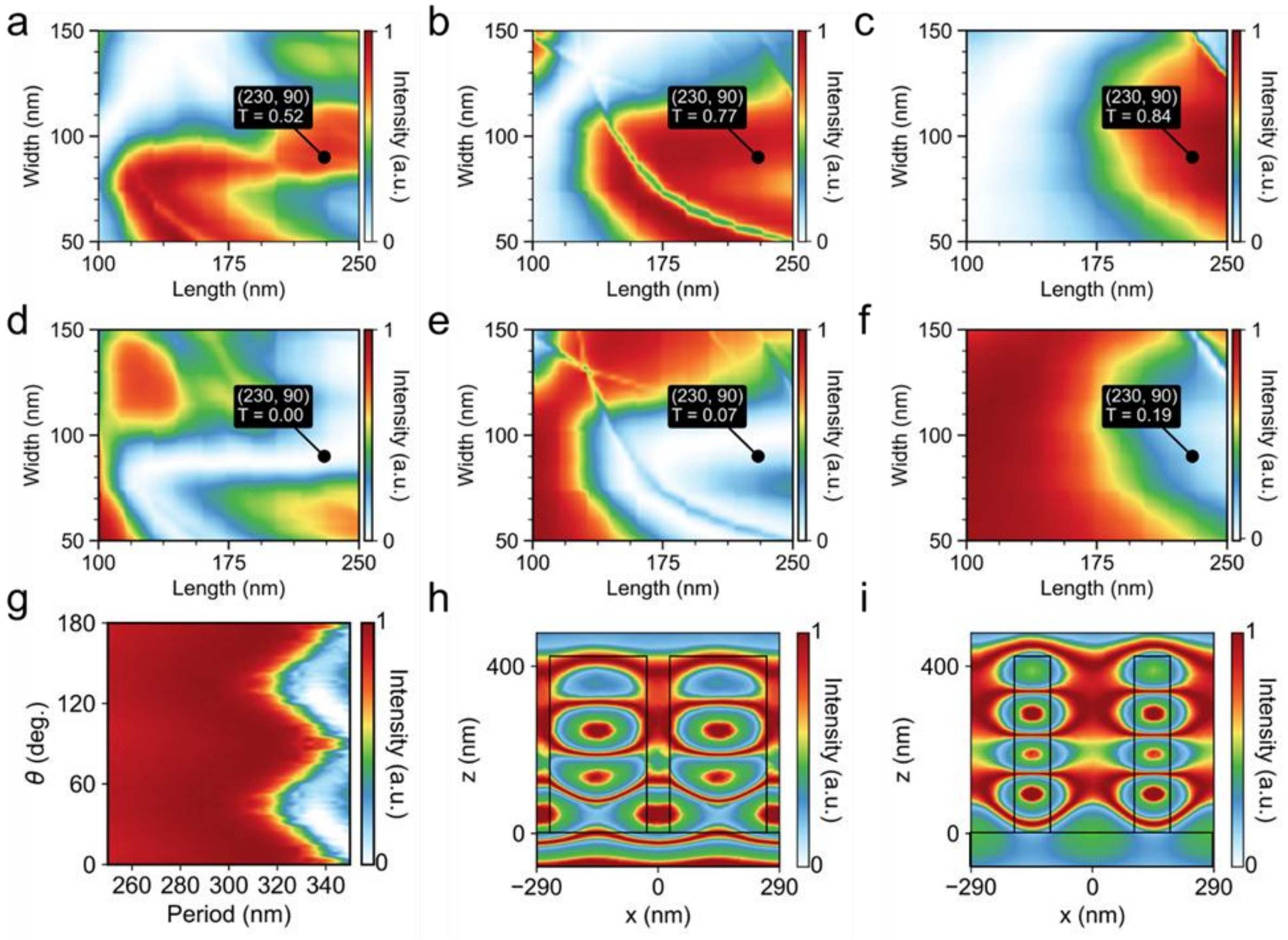

Figure S2: Multi-wavelength optimization and electromagnetic field distribution analysis of the a-Si:H metasurface unit cell. (a-c) Parametric transmission maps illustrate the spectral dependence of the cross-polarized transmission (T_{cr}) as a function of the unit cell width (W) and length (L) at wavelengths of 488 nm, 532 nm, and 633 nm. The optimized configuration (W = 90 nm, L = 230 nm) is marked by a black dot, achieving transmission efficiencies of T_{cr} = 0.52, 0.77, and 0.84, respectively. (d–f) Corresponding maps for the co-polarized transmission (T_{co}) under identical conditions, showing substantially reduced transmission (T_{co} = 0.00, 0.07, and 0.16), thereby confirming high polarization conversion efficiency and strong channel isolation. (g) Transmission response of meta-atom as function of in-plane orientation and lattice period (P). (h–i) Cross-sectional electric field intensity distributions ($|E|^2$) in the $x - z$ plane at the optimized geometry, revealing distinct resonance modes and localized field confinement responsible for the observed polarization-dependent response. Figures S2(h) and S2(i) correspond to the TE and TM excitation cases, respectively.

Section III: Numerical and analysis of focal position

The on-axis intensity distribution at an arbitrary point P with relative position p for metalens with stationary phase can be expressed as:

$$\mathbb{I}(\mathbf{0},\mathbf{0},\boldsymbol{z}) = |\boldsymbol{\mathcal{F}}_{\boldsymbol{z}}(\mathbf{0},\mathbf{0},\boldsymbol{z}).\boldsymbol{\mathcal{F}}_{\boldsymbol{z}}^{*}(\mathbf{0},\mathbf{0},\boldsymbol{z})| = [\boldsymbol{\mathcal{C}}^{2}(\boldsymbol{\gamma}) + \boldsymbol{\mathcal{S}}^{2}(\boldsymbol{\gamma})] \times \left(\frac{\mathbf{2}\boldsymbol{T}}{\boldsymbol{p}}\right)^{\mathbf{2}} \qquad \textbf{(SE1)}$$

Where $\mathcal{C}(\gamma) = \int_0^\gamma cos\left(\frac{\pi}{2}u^2\right)du$ and $\mathcal{S}(\gamma) = \int_0^\gamma sin\left(\frac{\pi}{2}v^2\right)dv$ are the Fresnel cosine and sine integrals, respectively. The parameter γ is the upper integration limit, given by $\sqrt{\frac{2|p|N}{1-|p|}}$ where N is the Fresnel number defined as $\frac{D^2}{4\lambda f}$ for square planner metalenses. While the relative axial position p is given by $\frac{1.4641}{2N+1.4641}$. Therefore, for metalenses with large longitudinal depth $(i.e.D \ll 2f)$, the position of maximum on-axis intensity p_m for a fixed-NA metalens and a preset focal length f with can be expressed as:

$$\boldsymbol{p_m} = \boldsymbol{f} - \boldsymbol{pf} \qquad \textbf{(SE2)}$$

This relation indicates that the axial intensity distribution can be then tuned by adjusting the aperture diameter D_a. It is worth noting that p is always positive, indicating that the positional shift occurs toward the metalens.

In order to more closely examine the dependence of the maximum intensity position $\boldsymbol{p_m}$on the aperture size, the optical responses of metalenses with different diameters were first investigated numerically. A detailed analysis is presented in **Table S1**, where the theoretically predicted positions and measured values of p_m confirm the effective application of classical Fresnel focal shift (CFF). It is worth noting that when the aperture size is reduced to half of the metalens diameter, the deviation between the predicted and measured positions becomes maximal due to pronounced edge diffraction effects. However, CFF based results for our metalens design greatly deviated from the predicted one because the distributed phase profile.

Table S1. Focal position analysis with CFF theory

**Type**	**Metalens Diameter (µm)**	**Aperture Diameter D_a (µm)**	**Preset Focal Position f (µm)**	**Fresnel Number N**	**Relative Position Shift p (%)**	**Predicted Focal Position p_m (µm)**	**Measured Focal Position (µm)**	**Deviation (%)**
	10		14.158	3.54	17.1	11.73	11.9	1.4
	15		21.237	5.31	12.1	18.66	18.4	1.4

	20		28.316	7.08	9.4	25.66	24.8	3.4
Simple	25		35.395	8.85	7.6	32.69	31.1	4.9
							**Mean Deviation (%)**	**2.7**
	20	10	28.316	1.77	29.3	20.03	19.9	0.6
		15	28.316	3.98	15.5	23.91	23.6	1.3
		17.5	28.316	5.42	11.9	24.94	24.3	2.6
							**Mean Deviation (%)**	**1.5**
Our metalens	10		14.2	3.54	17.1	11.73	6	48.8
	15		21.2	5.31	12.1	18.66	14.7	21.2
	25		35.39	8.85	7.6	32.69	23.5	28.1
	40		53	15.18	4.6	50.5	56.2	11.3
							**Mean Deviation (%)**	**27.35**
	20	10	28.316	1.77	29.3	20.03	15.14	24.4
		15	28.316	3.98	15.5	23.91	23	3.8
		17.5	28.316	5.42	11.9	24.94	27.4	9.9
	40	20	53	3.79	16.2	44.4	27.19	38.8
		30	53	8.54	7.9	48.8	41.7	14.5
							**Mean Deviation (%)**	**18.2**

Table S2. Focal position characterization using proposed model of our designed metalens

**Metalens Diameter (µm)**	**Aperture Diameter D_a (µm)**	f_{min} **(µm)**	f_{dof} **(µm)**	$f_{Predicted}$ **(µm)**	f_{Measured} **(µm)**	**Deviation (%)**
			**Simulations**			
5		1.25	5.8	7.1	6	15.2
10		2.5	11.7	14.2	14.7	3.8
15		3.75	17.5	21.2	23.5	10.7
					**Mean Deviation (%)**	**9.9**
20	10	5	23.3	16.7	15.2	8.8
	15	5	23.3	22.5	23.2	3.2
	17.5	5	23.3	25.4	27.4	7.9
	20	5	23.3	28.3	32.1	13.4
40	40	10	43.0	53.0	56.2	15.2
	30	10	43.0	42.3	41.7	1.3
	20	10	43.0	31.5	27.2	13.7
					**Mean Deviation (%)**	**7.75**

Section IV: Numerical analysis of metalens

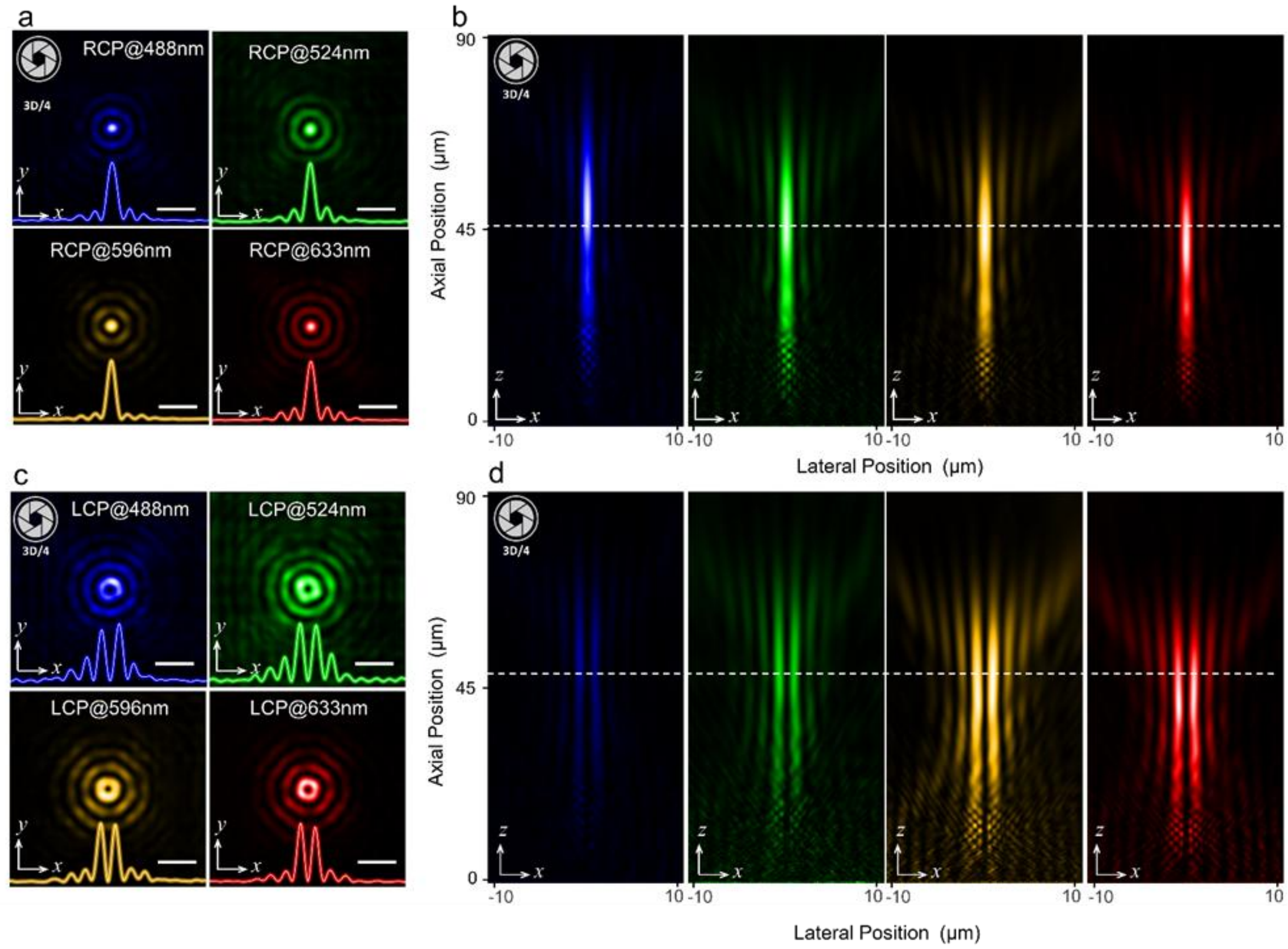

Figure S3: Effect of reduced aperture (3D/4) on the metalens focusing performance and maximum intensity position. (a, c) Transverse focal-plane intensity distributions (x–y plane, 10 μm × 10 μm) under RCP and LCP illumination, respectively, with the effective aperture reduced to 3D/4. RCP illumination produces a broadened Airy PSF with slightly enhanced side lobes, while LCP illumination generates a doughnut-shaped vortex with preserved 2π azimuthal phase accumulation, characteristic of a first-order orbital angular momentum (OAM) mode. (b, d) Corresponding longitudinal cross-sections along the optical axis (x–z plane, 10 μm × 90 μm), showing a modest broadening of the focal waist compared to the full-aperture case. It is worth noting that axial maximum intensity position deceases compared to full aperture illumination.

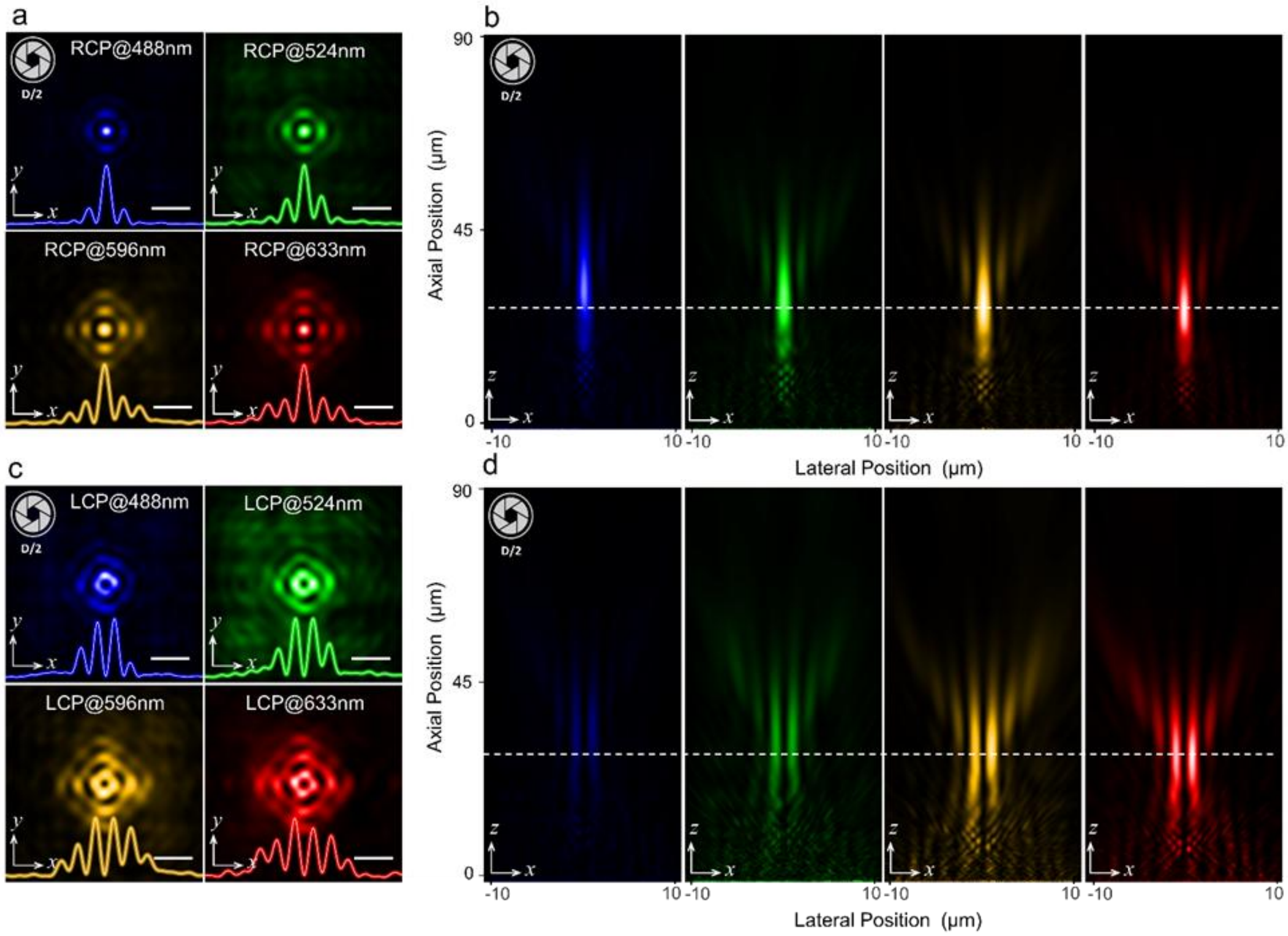

Figure S4: Effect of reduced aperture (D/2) on the metalens focusing performance and maximum intensity position. (a, c) Transverse focal-plane intensity distributions (x–y plane, 10 μm × 10 μm) under RCP and LCP illumination, respectively, with the effective aperture reduced to D/2. RCP illumination produces a noticeably broadened Airy PSF with more pronounced side lobes, while LCP illumination generates a doughnut-shaped vortex with preserved 2π azimuthal phase accumulation, characteristic of a first-order orbital angular momentum (OAM) mode. (b, d) Corresponding longitudinal cross-sections along the optical axis (x–z plane, 10 μm × 90 μm), showing further widening of the focal waist compared to the D and 3D/4 cases. Despite the increased side-lobe intensity and PSF broadening, the axial maximum intensity position deceases compared to former cases.

Section V: Fabrication and characterization and experimental Analysis

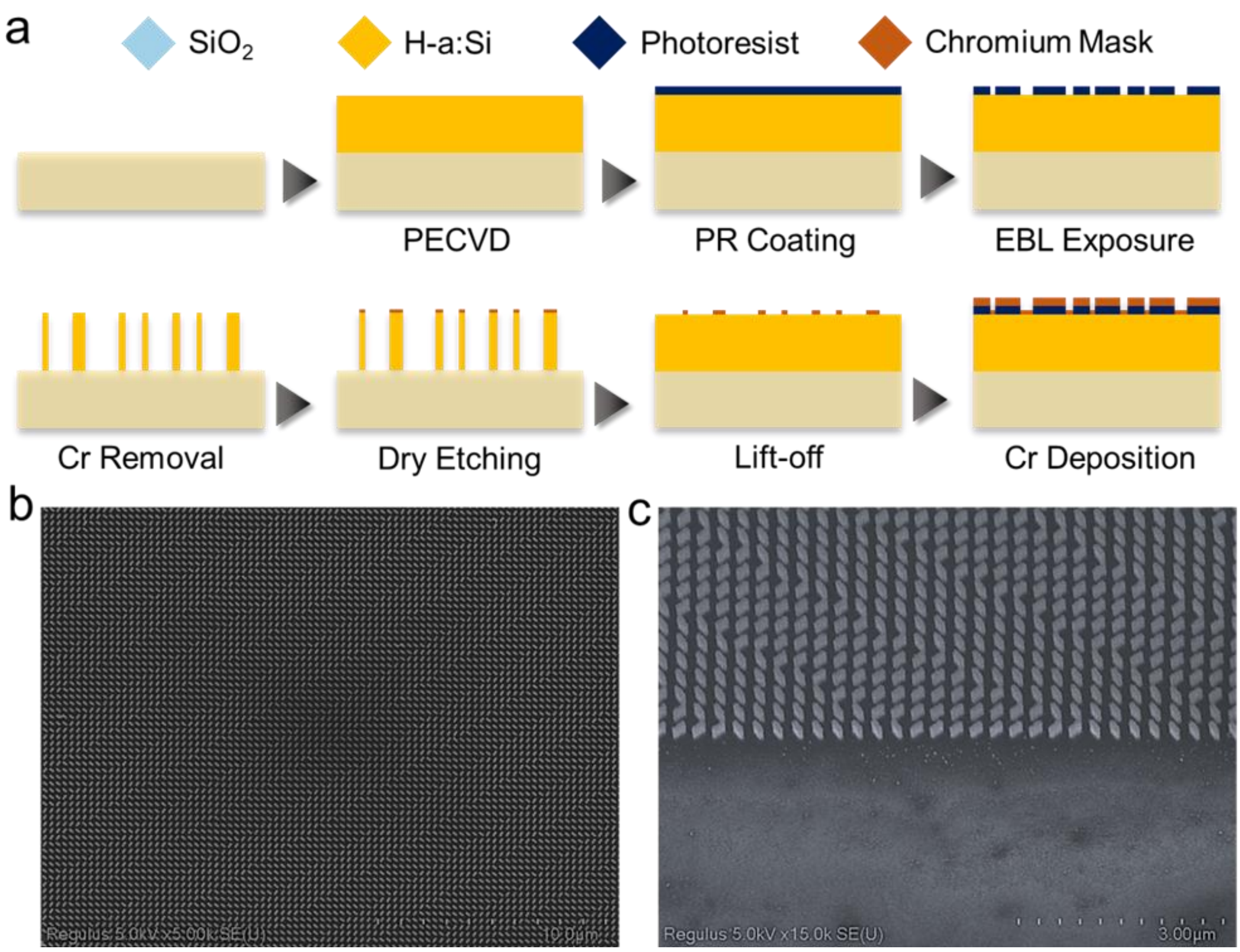

Figure S5. (a) Step-by-step fabrication workflow of the 1 mm × 1 mm metalens using electron-beam lithography (EBL), plasma-enhanced chemical vapor deposition (PE-CVD), and reactive ion etching (RIE). (b, c) SEM images of the fabricated metalens showing large-area uniformity and precise meta-atom orientation. Insets in (b) and (c) provide high-magnification views at 10 µm and 3 µm scales, respectively, highlighting the geometric fidelity of the nanostructures.

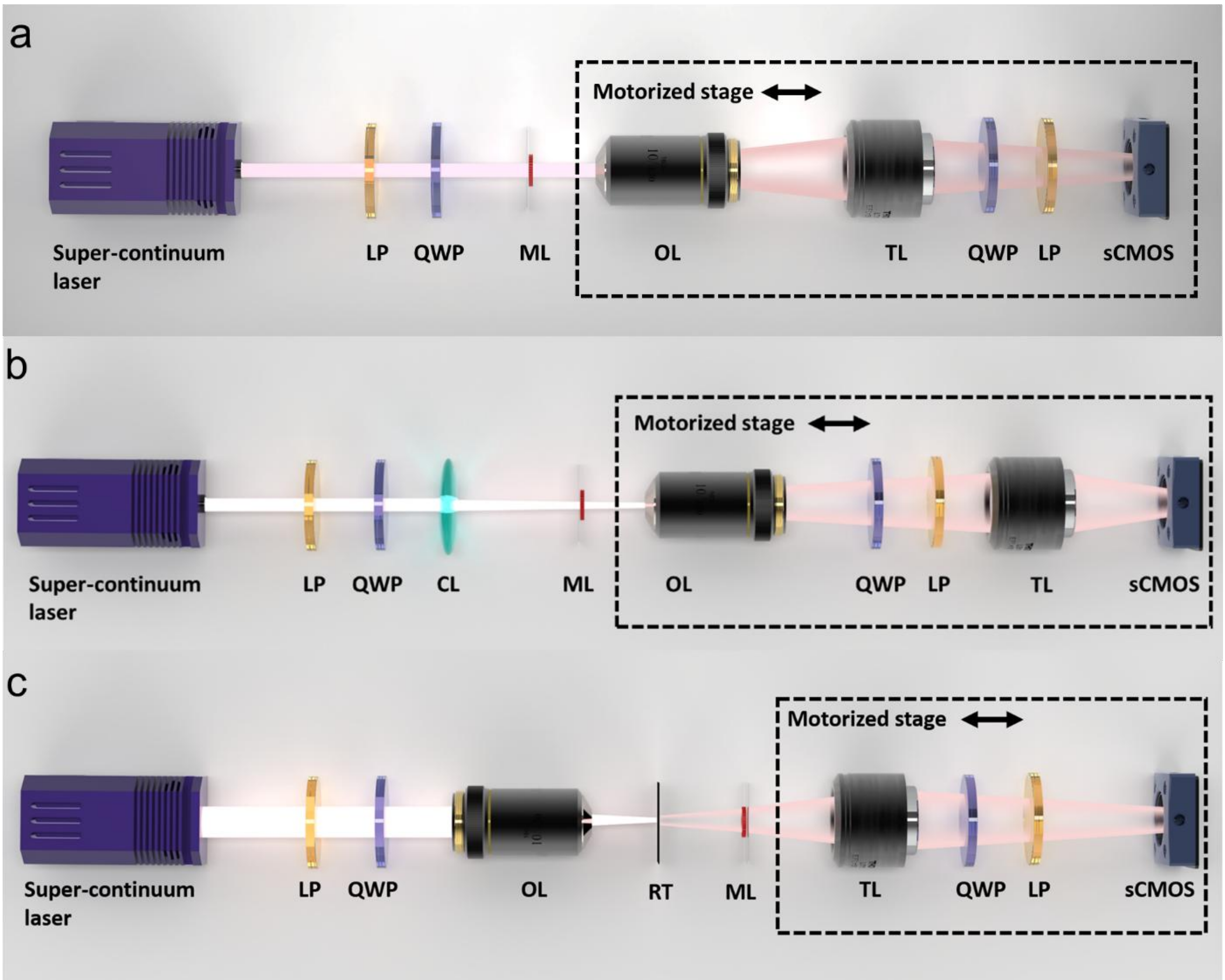

Figure S6: Figure S6. Schematic of the experimental setups used for polarization-resolved characterization of the achromatic metalens. (a) Optical configuration for full-aperture illumination (D), where light from a supercontinuum laser was spectrally filtered to the desired wavelength (488, 510, 532, 555, 575, 598, 610, or 633 nm), linearly polarized using a linear polarizer (LP), and converted to circular polarization by a quarter-wave plate (QWP) before illuminating the metalens (ML). (b) Optical configuration for reduced-aperture illumination (D/4), in which a convex lens (CL) was introduced before the metalens to reduce the effective beam diameter incident on the device. (c) Optical configuration for imaging the resolution target (RT). In all configurations, the transmitted field was collected by a high-numerical-aperture objective lens (OL) mounted on a motorized translation stage for axial scanning, analyzed using a QWP–LP polarization analyzer, and imaged onto an sCMOS camera through a tube lens (TL).

Section VI: Performance analysis

Table S3. Comparison of the proposed metalens with simple hyperbolic metalens.

**Type**	**KPI / $\lambda_{inci}(nm)$**	**488**	**510**	**532**	**554**	**576**	**598**	**610**	**633**
**Our Designed Metalens**	$Focal\ plane$(µm)	27.61	27.29	27.69	28.50	27.02	25.72	24.49	24.44
	$Focal\ Shift$ (µm)	0.58	0.90	0.5	0.31	1.17	2.47	3.69	3.75
	DoF	22.97	23.61	22.89	21.79	21.21	20.43	20.50	19.60
**Hyperbolic Metalens**	$Focal\ plane$(µm)	25.39	24.91	24.59	23.57	22.96	22.36	21.96	21.47
	$Focal\ Shift$ (µm)	2.79	3.28	3.59	4.62	5.23	5.83	6.23	6.72
	DoF	5.29	5.27	5.18	4.89	4.87	4.77	4.71	4.67

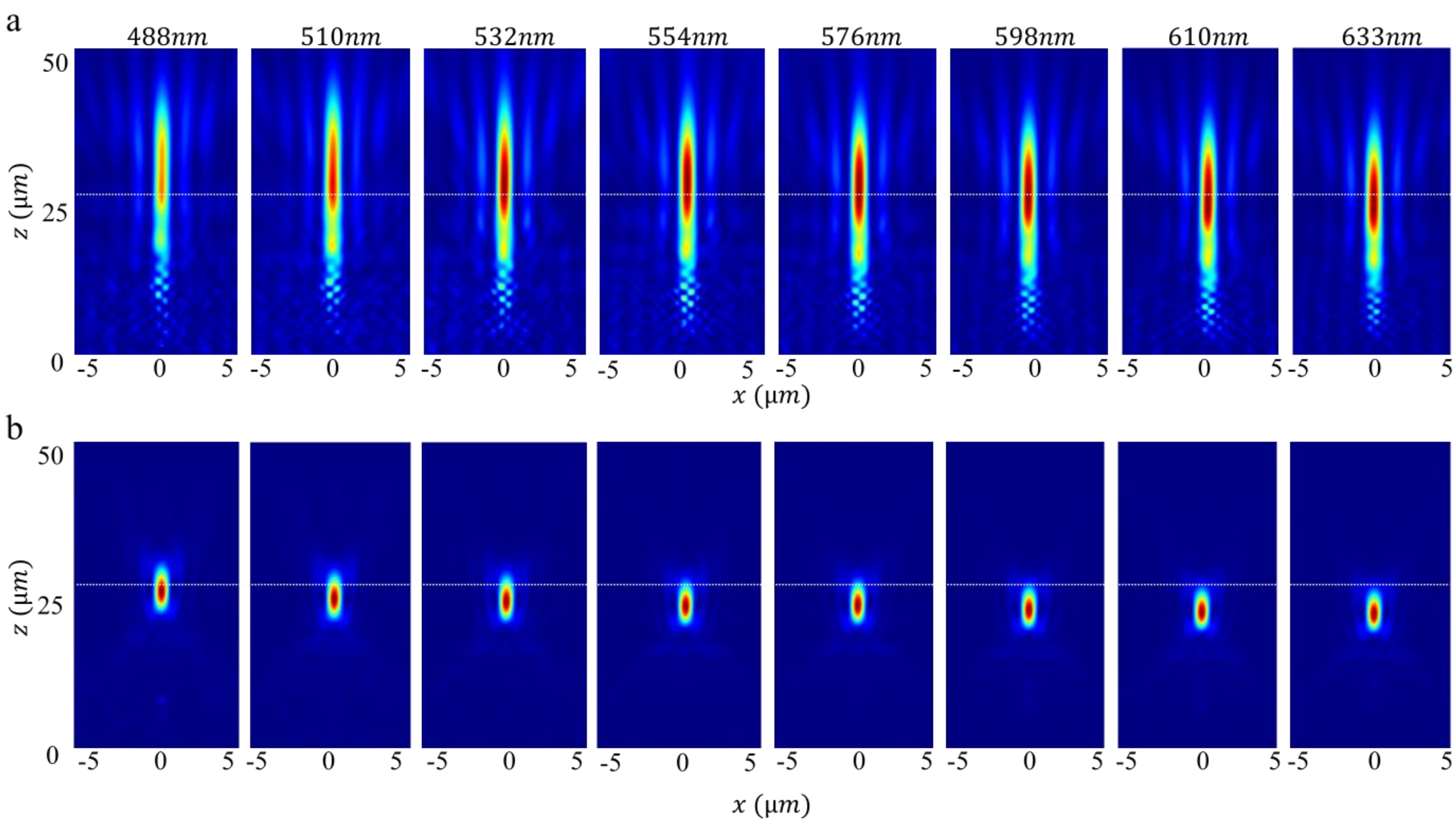

Figure S7. Comparison of the axial focusing performance of the proposed metalens and a conventional hyperbolic metalens, both designed for a wavelength of 560 nm with a NA of 0.33. (a) Simulated axial intensity distributions of the proposed metalens across the visible wavelength range from 488 to 633 nm. (b) Corresponding axial intensity distributions of the hyperbolic metalens over the same wavelength range. The proposed metalens maintains a DoF and exhibits superior achromatic focusing performance across the visible spectrum compared with the conventional hyperbolic metalens, while operating at the same design wavelength and numerical aperture.

Table S4. Comparison of the proposed metalens with representative achromatic metalenses.

Reference	Bandwidth	Polarization Multiplexing	Focal Position Control	Functionality	NA
Chen et al., 2018. [1]	470-670 nm	✗	✗	Broadband achromatic imaging	0.20
Chen et al., 2019. [2]	460-700 nm	✗	✗	Broadband achromatic imaging	0.20
Ndao et al., 2020. [3]	640-1200 nm	✗	✗	Ultra-broadband achromatic focusing	0.12
Hu et al., 2023. [4]	400-1000 nm	✗	✗	Ultra-broadband achromatic focusing	0.164
Chang et al., 2024. [5]	400-700 nm	✗	✗	Full-visible achromatic imaging	0.02
Lu et al., 2024. [6]	9000-12000 nm	✓	✗	Broadband achromatic polarization-multiplexed metalens	0.34
Du et al., 2025. [7]	450-700 nm	✓	✗	Full-color quasi-achromatic dual-functional imaging	0.2
This work	488-633 nm	✓ (LCP/RCP)	✓	Broadband bright-field / edge-enhanced dual-mode imaging	≈0.33

References

[1]. Chen, W.T., Zhu, A.Y., Sanjeev, V. et al. A broadband achromatic metalens for focusing and imaging in the visible. Nature Nanotech 13, 220–226 (2018). https://doi-org/10.1038/s41565-017-0034-6

[2]. Chen, W.T., Zhu, A.Y., Sisler, J. *et al.* A broadband achromatic polarization-insensitive metalens consisting of anisotropic nanostructures. *Nat Commun* **10**, 355 (2019). https://doi-org/10.1038/s41467-019-08305-y

[3]. Ndao, A., Hsu, L., Ha, J. *et al.* Octave bandwidth photonic fishnet-achromatic-metalens. *Nat Commun* **11**, 3205 (2020). https://doi-org/10.1038/s41467-020-17015-9

[4]. Hu, Y., Jiang, Y., Zhang, Y. *et al.* Asymptotic dispersion engineering for ultra-broadband meta-optics. *Nat Commun* **14**, 6649 (2023). https://doi-org/10.1038/s41467-023-42268-5

[5]. Chang, S., Zhang, L., Duan, Y. *et al.* Achromatic metalenses for full visible spectrum with extended group delay control via dispersion-matched layers. *Nat Commun* **15**, 9627 (2024). https://doi-org/10.1038/s41467-024-53701-8

[6]. Lu, Yilin, Ruohui Chen, Chao Hu, Xiaofei Liu, Zhikai Gan, Xingyan Zhao, Yang Qiu et al. "All-silicon metalens for broadband achromatic polarization multiplexing in long-wave infrared wavelengths." *Applied Optics* 63, no. 12 (2024): 3242-3249. https://doi.org/10.1364/AO.520895

[7]. Du, Xiaomin, Wenjing Shen, Jian Li, Yanhao Chu, Jiacheng Sun, Junyi Wang, Jitao Ji, Chen Chen, Shining Zhu, and Tao Li. "Full-color quasi-achromatic imaging with a dual-functional metasurface." *Nano letters* 25, no. 20 (2025): 8143-8150. https://doi.org/10.1021/acs.nanolett.5c00695
